# On the Effects of Disordered Tails, Supertertiary Structure and Quinary Interactions on the Folding and Function of Protein Domains

**DOI:** 10.3390/biom12020209

**Published:** 2022-01-26

**Authors:** Francesca Malagrinò, Valeria Pennacchietti, Daniele Santorelli, Livia Pagano, Caterina Nardella, Awa Diop, Angelo Toto, Stefano Gianni

**Affiliations:** Istituto Pasteur—Fondazione Cenci Bolognetti, Dipartimento di Scienze Biochimiche “A. Rossi Fanelli” and Istituto di Biologia e Patologia Molecolari del CNR, Sapienza Università di Roma, 00185 Rome, Italy; francesca.malagrino@uniroma1.it (F.M.); valeria.pennacchietti@uniroma1.it (V.P.); daniele.santorelli@uniroma1.it (D.S.); livia.pagano@uniroma1.it (L.P.); caterina.nardella@uniroma1.it (C.N.); awa.diop@uniroma1.it (A.D.)

**Keywords:** multidomain proteins, IDPs, crowding, protein–protein interactions

## Abstract

The vast majority of our current knowledge about the biochemical and biophysical properties of proteins derives from in vitro studies conducted on isolated globular domains. However, a very large fraction of the proteins expressed in the eukaryotic cell are structurally more complex. In particular, the discovery that up to 40% of the eukaryotic proteins are intrinsically disordered, or possess intrinsically disordered regions, and are highly dynamic entities lacking a well-defined three-dimensional structure, revolutionized the structure–function paradigm and our understanding of proteins. Moreover, proteins are mostly characterized by the presence of multiple domains, influencing each other by intramolecular interactions. Furthermore, proteins exert their function in a crowded intracellular milieu, transiently interacting with a myriad of other macromolecules. In this review we summarize the literature tackling these themes from both the theoretical and experimental perspectives, highlighting the effects on protein folding and function that are played by (i) flanking disordered tails; (ii) contiguous protein domains; (iii) interactions with the cellular environment, defined as quinary structures. We show that, in many cases, both the folding and function of protein domains is remarkably perturbed by the presence of these interactions, pinpointing the importance to increase the level of complexity of the experimental work and to extend the efforts to characterize protein domains in more complex contexts.

## 1. Introduction

The majority of the human proteome has a modular nature and is organized in building blocks denoted as protein domains [1]. It may be shallowly stated that small proteins (i.e., smaller than 100 amino acids) are often found as single-domain units, whereas larger systems tend to consist of multiple domains. Thus, the complex assembly of different structural elements may account for achieving multiple combined cellular functions that are commonly played by large proteins.

It is a general belief that protein domains may fold, function and evolve independently from the remainder of the protein [2]. Moreover, it is very frequently observed that protein domains can be successfully expressed in isolation, and they tend to fold spontaneously into their native three-dimensional structure while maintaining their overall functions. Due to the complexity of studying larger proteins using standard biophysical techniques, it is, therefore, very common to investigate the structural and functional properties of isolated domains. In these cases, general rules are then extracted by studying, in detail, the individual domains, while assuming that large proteins behave as beads-on-a-string, i.e., the function of a large protein can be understood by summing up the functions of its individual domains.

The discovery that a up to 40% of the proteome is intrinsically denatured, while fully functional, has considerably revolutionized our understanding of the protein universe [3,4,5,6,7,8,9]. For example, several studies have unambiguously shown that, in many cases, disordered protein segments, which can be hardly defined as ‘domains’, appear to be critical in modulating the functions of proteins and demand a rigorous experimental characterization [10,11,12,13,14,15,16,17,18,19]. This observation clearly complicates our view of protein structure and challenges the definition of protein domain, per se.

**Figure 1:**
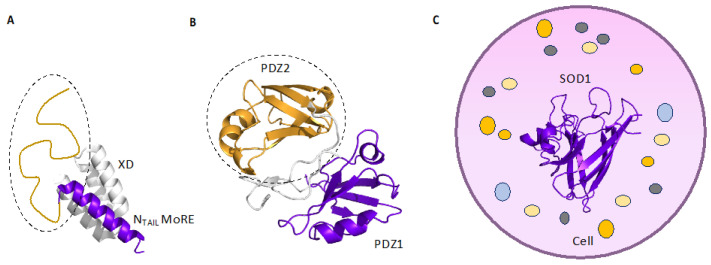
(**A**) Structural representation of the complex between the intrinsically disordered protein N_TAIL_ bound to the globular domain XD from measles virus. The αMoRE region of N_TAIL_ (in purple) undergoes a disorder-to-order transition upon binding with XD, with a flanking portion remaining disordered after binding (highlighted in orange). This flanking disordered region has been demonstrated to influence the folding properties of N_TAIL_ αMoRE. (**B**) Tandem of PDZ domains (PDZ1-PDZ2) of Whirlin protein. The effect of the presence of a denatured PDZ2 in the folding reaction of PDZ1 has been extensively studied, highlighting the formation of a misfolded intermediate, not detectable in experimental conditions, in which PDZ2 was held in its native conformation. This misfolded intermediate has been demonstrated to retain the physiological binding activity with a natural ligand of Whirlin. (**C**) Schematic representation of the interactions occurring between SOD1 protein and the crowded intracellular milieu, denoted as “quinary structure”. The folding mechanism of SOD1 protein (in purple) has been characterized *in cellula* and it has been found influenced by the intracellular environment.

Yet, how does a protein domain change when is studied in isolation as compared with more complex architectures? Whilst the role of quaternary structure in modulating the affinity and allostery of a protein domain has been well established since the pioneering work on hemoglobin and myoglobin [20], in this review we resort to recapitulating part of the emerging themes in the literature, highlighting the effects that are played by (i) flanking disordered tails; (ii) contiguous protein domains; (iii) interactions with the cellular environment, generally defined as ‘quinary structures’ (exemplified in Figure 1). In an effort to offer a comprehensive view on this matter, we focus on the effects that are produced in protein domains, both from a folding and from a functional perspective.

## 2. Disordered Tails

A large fraction of the human proteome is characterized by the presence of intrinsically disordered segments, whose length may vary considerably, and which may account for up to 40% of expressed proteins [3,4,5,6,7,8,9]. In the case of multi-domain proteins, for example, it is frequently observed that folded domains are interconnected by disordered stretches. Interestingly, these elements do not solely represent a passive physical connection between the functional domains but appear to play a more active role. From a folding perspective, it is important to distinguish the analysis of the cases in which disordered segments represents a physical interconnection between folded elements, from other cases in which relevant effects have been addressed in the peripheral peptidic segments of protein domains.

In the case of multi-domain proteins composed of several domains connected by disordered segments, a growing number of studies are supporting the view that these parts may be critical in ensuring cooperative folding of multi-domain proteins [21,22,23,24,25]. In fact, because of their inherent flexibility, they may modulate the communication between non-adjacent robust domains, thus ensuring the formation of stable folding nuclei that culminate with the observed cooperative folding of the whole multi-domain protein [22,23,24,25,26]. In these cases, disordered segments act as physical connectors between different folding nuclei, thus optimizing the productive collision between embryonic native structure formation. These findings suggest that disordered segments, displaying a high flexibility, may show an unrecognized relationship with folding and unfolding kinetics [27,28]—a hypothesis that was subsequently addressed directly and confirmed [29].

Whilst it is clear that the physical interconnection between structured elements might be critical in regulating their interaction and folding, the effect of disordered tails, acting as appendages of folded domains, is somehow more complex. In this context, an interesting case is exemplified by the study of the interaction between the nucleoprotein and phosphoprotein of measles [5,30,31,32]. In this case, the binding is mediated by a folded domain of the phosphoprotein, denoted XD, and a short C-terminal segment of the nucleoprotein, known as N_TAIL_ MoRE, which is intrinsically unstructured in isolation but undergoes a disorder-to-order transition upon binding, leading to an alpha-helical structure [5,6,10,30,31,32]. Notably, whilst the analysis of the NMR structure between XD and N_TAIL_ would suggest that the latter protein interacts physically with XD only via its MoRE [5], additional studies have proven a more complex scenario. In fact, by systematically truncating the disordered region flanking the MoRE while monitoring the effect on the folding and unfolding rate constants [33,34,35], it was possible to demonstrate that the disordered region has a pronounced effect on the folding mechanism, with longer variants displaying a slower folding rate constants than the shorter ones. In theory, such effects may either arise from steric hindrance brought by the disordered extension or from the direct interaction between specific residues. Remarkably, site-directed mutagenesis designed on a position located in the disordered tail highlighted some specific residues that were capable of also increasing the folding rate constant of the MoRE in the case of the full-length N_TAIL_ [35], suggesting that there is a direct role of specific side chains within the flanking disordered tail in the self-inhibition of folding. Of additional interest, a comprehensive in vitro and *in cellula* analysis of the truncation variants indicated that the presence of a highly dynamic disordered segment in N_TAIL_ decreases its binding affinity for XD [36], thereby modulating their interaction in a physiological context. Thus, the transient interactions between the MoRE and the fuzzy appendage appear to favor the disordered conformation, thereby tuning down the affinity between N_TAIL_ and XD to match physiological requirements.

Aside from their effects on folding, one of the possible scenarios whereby the presence of contiguous disordered tails may modulate the functional properties of folded domain is represented by the direct interaction with the substrate. For example, in the case of protein–DNA binding, the specific recognition event is primarily assigned to the contacts between the interacting domains and the bases or phosphates of the DNA [37]. Interestingly, however it has been observed that also the residues of contiguous disordered segments may modulate binding affinity [38,39]. These regions, while lacking a folded structure may form transient interactions with the DNA target, thereby influencing the overall affinity. An example of this type of scenario is represented by the so called fly-casting mechanism [39]. By following this view, the presence of an increased capture radius by intrinsically disordered systems is thought to optimize DNA binding. The analysis of the mechanisms whereby homeodomains recognize their specific DNA target sequence provided a clear-cut support of this view [40,41]. On the basis of these findings, the existence of a dynamic DNA readout mechanism was proposed, whereby distant segments modulate conformational preferences, flexibility or spacing of the DNA binding motifs or serve as competitive partners [42]. Thus, in these cases, it appears that contiguous disordered tails may directly interact with physiological partners via interactions that are not obvious from the sole analysis of static three-dimensional structure which, therefore, requires a detailed study that also infers the dynamics and flexibility of the overall complex. 

The role of flanking disordered tails in modulating the functions of neighboring domains is not solely limited to their capability to increase or decrease the affinity for the partners. In fact, in some cases, a documented effect may be ascribed to the fine-tuning of the allosteric properties of the contiguous domains, via mechanisms that are even more difficult to capture. The human enzyme UDP-α-d-glucose-6-dehydrogenase catalyzes the NAD+-dependent oxidation of UDP-α-d-glucose (UDP-Glc) to UDP-α-d-glucuronic acid. The enzyme contains a 30-residue disordered C-terminal segment that has been classically cleaved with no relevant effects on the kinetic parameters of the enzyme [43], leading to the view that the presence of the disordered tail flanking the enzyme had no effects on its functions. Nevertheless, a recent investigation on the allosteric properties of UDP-α-d-glucose-6-dehydrogenase revealed that the entropic force produced by an intrinsically disordered carboxy-terminus has a pronounced effect on the energy landscape of the enzyme [44]. In particular, it was observed that the disordered tail is capable to dictate the population of a sub-state with a high affinity for an allosteric inhibitor. Remarkably, the function of such disordered tail seems not to depend on its sequence or chemical composition, but solely on its length. Similar effects were also observed in the case of the αα-hub domain from radical induced cell death1 [45]. These findings suggest that the size of disordered tails flanking functional enzymes might be under evolutionary pressure and might be critical in fine-tuning their allosteric properties.

## 3. Interaction between Protein Domains—Supertertiary Structure

The architecture of proteins is often composed by several protein domains playing different roles that account for the multi-faceted functions of large proteins. Whilst it is very frequent that isolated domains fold independently from the remainder of the protein and maintain some of their physiological properties, it is very important to define the energetic communication between such building blocks that contributes to their physiological role. 

From a thermodynamic perspective, our comprehension of multi-domain systems is generally very limited. In fact, only in a few cases, the stability of isolated protein domains could be compared with those observed in more complex constructs. A systematic analysis of the interactions between type III domains within the cell-binding region of fibronectin showed that contiguous domains might affect their stability both by stabilizing and by destabilizing interactions [46]. Moreover, a more surprising observation revealed that in some cases the interactions between domains display a cooperative nature, such that two consecutive domains may fold as a single co-operative unit. This finding could be subsequently observed, also, on non-homologous domains [47], leading to the view that drawing general rules assuming protein domains as independent folding units are unlikely and reinforcing the importance of studying, directly, the folding of multi-domain proteins. 

The complexity of the molecular architecture of multi-domain systems is also mirrored by their folding behavior. From this perspective, it is particularly informative to analyze the few cases in which the behavior of a multi-domain system could be directly compared with its isolated domains. Furthermore, it is worth differentiating the effects that have been highlighted in cases in which (i) two contiguous domains are both denatured and (ii) only one of the domains is denatured, whereas the other one is held in its native conformation. In fact, a number of studies have shown that the simultaneous denaturation of two contiguous domains may have profound effects on their folding, which include the accumulation of misfolded intermediates that compete with and remarkably slow down productive folding. By analyzing the folding kinetics of tandem repeats [48,49,50,51], it was shown that the tendency to form such misfolded conformations increases with increasing sequence homology of contiguous domains [52], strongly suggesting that these misfolded conformations involve transient domain swapping [53]. In line with these observations, a bioinformatic analysis of the human proteome revealed that the sequence identity between contiguous homologous domains tend to be lower than 40% [52]. This finding suggested the presence of an evolutionary pressure to keep such value at a low level and, therefore, minimize misfolding and aggregation events. Remarkably, however, in the case of the tandem repeat involving the first two PDZ domains of whirlin, the transient misfolding intermediate arising from the concurrent denaturation of the two PDZ domains maintains its capability to interact with its physiological ligand [51]. This finding suggests that transient misfolding of tandem repeats may display a hidden physiological role that demands additional investigation.

The effect on the folding of a protein domain in the absence and in the presence of a contiguous native domain could be explored extensively in the case of the whirlin [54]. In fact, in this case, the fortuitous difference in stability between the two PDZ domains allowed performing a complete mutational work on in the context of the tandem, while keeping the second PDZ domain, which is denatured only at very high denaturant concentrations, in its native state. Such mutational work was then compared with the same approach performed on PDZ1 in isolation [54]. On the basis of this analysis, it could be concluded that whilst the late events of folding are robust, the early events are much more malleable and display clear structural changes. Interestingly, such changes superpose with the frustration patterns of the domain, suggesting that, in multi-domain folding, alternative pathways, and possibly protein misfolding, may arise from locally non-optimized regions, which appear to be mainly involved in protein function.

Yet, what is the effect of inter-domain interactions in the function of proteins? One of the first studies to analyze, in detail, the physiological properties of a multi-domain protein based on the dynamics of inter-domain interactions in a multi-domain system was contributed by McCann et al., who employed single-molecule fluorescence resonance energy transfer to describe the structure of the scaffold protein PSD-95. [55]. By using this method, the authors proposed the protein to behave as partitioned between two independent subunits comprising the first two PDZ domains (PDZ1 and PDZ2) and the PDZ3-SH3-GK (PSG) domains respectively. Such behavior was subsequently confirmed by a comprehensive analysis based on the use of nuclear magnetic resonance, small-angle X-ray scattering and Rosetta modelling [56]. Accordingly, the tight interaction between the individual domains within these supramodular structures was denoted as ‘supertertiary structure’ and the communication between the SH3 and PDZ3 domains was demonstrated to have a direct effect on the affinity of PSD-95 for its physiological ligands [57].

The inter-domain energetic communication within the PSG supramodule was also investigated by double mutant cycles [58]. The so-called ‘double mutant cycles’ methodology is a powerful approach to measuring, directly, the extent of energetic communication between interacting side-chains. The approach is founded on the synergic employment of site-directed mutagenesis and quantitative measurement of the biophysical properties of a protein system [59,60,61,62]. A double-mutant cycle postulates that mutations insisting on two non-interacting residues would return an additive effect. Thus, in this case, a double mutant displaying the two perturbations is expected to display the effects of the sum of the two mutations measured individually. Conversely, in the case of interacting residues, the free energy of interaction may be measured by comparing the effect of the perturbation on the double mutant, arising either from a structural or functional property of the protein, with respect to that probed on the single mutants. The power of the double mutant cycle lies in the possibility of measuring the free energy of coupling between residues regardless any structural knowledge. Hence, by treating the system as a ‘black box’, couples of interaction energies may be promptly obtained simply by performing site-directed mutagenesis while measuring the structural and functional property of the protein. 

**Figure 2:**
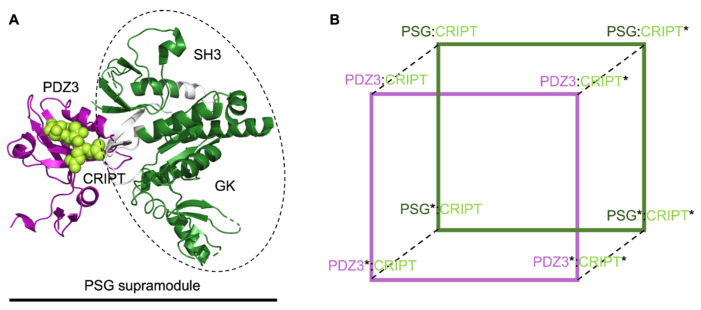
(**A**) Structural representation of the bound complex between the PSG supramodule (PDZ3-SH3-GK domains) of PSD-95 protein and a peptide mimicking a specific portion of the CRIPT protein. The binding between the PDZ3 domain (in purple) and CRIPT (in light green, spheres) was characterized in the presence and in the absence of the two neighboring SH3 and GK domains (in dark green). (**B**) Double-mutant cycle of the binding of PDZ3 with CRIPT was performed both on PDZ3 in isolation (purple square, PDZ3:CRIPT) and in the PSG supramodule (green square, PSG:CRIPT). By analyzing the changes in thermodynamic parameters of the binding upon mutations (highlighted with a *) on CRIPT (PDZ3/PSG:CRIPT*), on PDZ3 in the PSG supramodule (PDZ3/PSG*:CRIPT) and on both the proteins (PDZ3/PSG*:CRIPT*) it has been possible to define the energetic allosteric network underlying the recognition and binding of PDZ3 with CRIPT within the PSG supramodule.

In the case of the PSG supramodule from PSD-95 (see Figure 2), it was unambiguously demonstrated by double-mutant cycles that the allosteric property of the PDZ domain in recognizing the physiological ligand is remarkably influenced by the presence of the contiguous domains [57]. This finding parallels the earlier observation that the presence of an additional helix in the PDZ domain may also affect its dynamic and allosteric properties [63]. On the basis of these observations, it was suggested that the sensitivity of allosteric networks to the presence of adjacent protein domains may represent a common property of supertertiary structures in proteins, therefore urging the literature to address the functional properties of protein domains in the context of their physiological architecture, rather than in isolation.

## 4. Protein Domains in the Crowded Cellular Environment—The Quinary Structure

Ever since the 1980s, it has been suggested that the interactions between the protein domains within the cellular environment could play a key role in their stability, folding and function [64,65]. These types of interactions, which are primarily mediated by proteins’ surfaces, were thought to be so important as to represent, de facto, an additional level of structural organization of proteins, denoted as the ‘quinary structure’ [64,66]. Despite this growing appreciation, because of the experimental difficulties in studying proteins in such a complex matrix, our current knowledge on the effects on the quinary structure is still limited, and may account on relatively few experimental studies. 

The effect on protein stability of quinary interactions has been studied in detail by double mutant cycles in the case of the B domain in protein G [67]. In particular, by mutating the protein while measuring its stability by hydrogen/deuterium exchange, it has been possible to compare the thermodynamic stability in isolation as compared with those measured in *E. coli* cell lysates. Interestingly, it has been observed that replacing surface residues displays very different effects in an isolated domain as compared to what could be measured in the cellular environment, suggesting that attractive interactions between the protein surface and the cytosol are critical in modulating the stability of the protein, even though the change has a negligible effect in dilute solution. This finding was also paralleled by other studies that successfully established how quinary interactions may either stabilize or destabilize different proteins when considered in the crowded cellular environment [68,69,70,71].

To further understand the effects of quinary interactions in the stability of a globular protein, Oliveberg, Selenko and co-workers have performed a detailed in-cell NMR study on a marginally stable variant of superoxide dismutase (SOD) [72]. In this work, the behavior measured *in cellula* was considered both in mammalian and bacterial cells and compared to that measured in vitro, in the absence and in the presence of different molecular crowders, displaying the theoretical ability to mimic the cellular environment. It was observed that both mammalian and bacterial cells not only destabilize SOD, but also have a pronounced effect on the heat capacity of (un)folding, narrowing the width of the thermal unfolding transitions. In this case, an increase in heat capacity results in a shift of both cold and heat denaturation into the physiological regime. Importantly, the transient quinary interactions appear sequence- and context-dependent, thus explaining why different proteins may display a different behavior when considered in their physiological conditions [68,69,70,71]. On the basis of these observations, it was concluded that proteins are optimized not only for their structure and function but also for their inherent capability to transiently interact with their specific host-cell environment. 

Whilst several studies successfully depicted the effects of the cellular environment on protein stability, the mechanism of folding and unfolding remains more elusive. Only recently, a successful characterization of the folding and unfolding kinetics of GB3 measured by in-cell NMR was reported [73]. In particular, by performing site directed mutagenesis on the small globular domain, it was possible to successfully improve the HSQC spectra of the protein, allowing characterization of both the protein structure and its folding and unfolding dynamics. The analysis demonstrated that, whilst the native structure of the protein is not affected in the cellular environment, its folding and unfolding kinetics appear perturbed. Importantly, however, as it could be predicted from in vitro studies, the mechanism of folding is very robust and is maintained in the cell, as probed by the conserved two-state nature of the reaction. More to the point, the authors showed that crowding agents, as well as lysate samples, could not efficiently reproduce the effects observed in the living cells [73]. This finding supports the view that quinary interactions, which are most likely perturbed in lysates, are critical, shaping the balance of forces regulating the folding of single-domain proteins. 

From a functional perspective, the existence of quinary interactions has been principally depicted in the concept of the ‘metabolon’. By following this view, the physical interaction between different enzymes in the same metabolic pathway is thought to enhance the enzymatic activity [65,74,75]. In fact, by considering that different enzymes in the same metabolic pathway led to the theoretical accumulation of non-functional intermediates, the association of such enzymes in supramolecular complexes minimizes the lifetime of such intermediates, therefore optimizing the metabolism, per se. The existence of such metabolons has been proven in several cases [65,76], leading to a detectable difference of the enzymatic activity measured in isolation as compared with that observed *in cellula*. In this context, it is particularly informative to exemplify the work focused on the enzymes participating in the Krebs cycles [77], which represent one of the most characterized group of enzymes participating in the formation of a metabolon complex. In this case, it has been demonstrated that the enzyme optimization occurs primarily by accelerating the reaction rates, minimizing the delay time for the enzymes and substrate to diffuse in solution. Other example of ‘substrate channeling’ involving metabolons have been demonstrated in the case of the enzyme hexokinase and glucose-6-phosphate dehydrogenase in the glycolytic pathway [78], as well as in the case of aminoacyl-tRNAs synthetase during protein synthesis [79]. 

Overall, it appears that the surface of proteins might be critical in the cellular context both from a structural and from a functional perspective. In fact, the surface mediated interactions might be very important in orchestrating several physiological transient binding events. These effects cannot be directly directed on isolated proteins and protein domain, thus suggesting that the functional and structural role of protein surfaces might have been overlooked and demand additional characterization.

## 5. Theory and Simulations

Due to the inherent difficulties in addressing complex multidomain systems using theoretical and molecular dynamics approaches, the definition of a unifying theory describing the effects of disordered tails, contiguous domains and quinary interactions on the folding and function of protein domain is still far from being achieved. Nevertheless, several groups have tried to date to address this very difficult question.

A recent work by Taneja and Holehouse investigated the combined role of folded domains and disordered tails in governing the conformational behavior of the disordered regions via all-atom molecular dynamics simulations [80]. These simulations were performed on different disordered tails attached to the superfolder green fluorescent protein. It was observed that emergent interactions between disordered tails and the domain were very complex and in large part unintuitive due to the conformational flexibility of tails, and the competing and long-range nature of electrostatic interactions that were formed by the disordered tails and the folded domains. These findings suggest that, as highlighted above, the complex crosstalk between disordered tails and contiguous folded domains may represent an additional and complex layer of conformational regulation of proteins and demands additional investigation.

Molecular dynamics simulation studies on multidomain proteins have recently attracted the attention of several groups and have been reviewed already, see, for example, [81,82]. In some relevant cases, a clear-cut difference in the folding and function of protein domains could be detected when comparing the domain in isolation and in more complex constructs. For example, hepatitis C protein 3/4A is a bifunctional enzyme consisting of two distinct domains that are devoted to the protease and helicase activities. These catalytic effects are critical for viral infection and propagation. Importantly, the activity of the individual domains was found to be different in the full-length protein [83]. In an effort to explain these effects, Schiffer and co-workers performed extensive molecular dynamics simulations [84]. It was found that the two domains are dynamically coupled via their respective interfaces. This coupling has a direct effect on the RNA unwinding activity of the enzyme, such that the full-length protein was found to be more efficient than the isolated protease domain. On the basis of these observations, it was suggested that the protein 3/4A may explore an extended catalytically active conformation, in which the interface between the two catalytic domains acts as a switch to regulate activity. 

Several simulations studies have investigated how cellular crowders may influence the folding and function of protein domains, as exemplified in [85,86,87,88,89,90]. As briefly recalled above, in these cases, it was observed that molecular crowders can both stabilize and destabilize proteins. Of particular interest, simulations performed on a realistic model of the bacterial cytoplasm including 50 of the most abundant proteins of *E. coli* have successfully described the diffusional and thermodynamic perturbations induced by the cell on the green fluorescent protein [91]. These types of studies represent a very interesting platform to contribute to our understanding of quinary interactions and their effect on folding and function. 

## 6. Conclusions

Thanks to the continuous development of different experimental and theoretical techniques, our current knowledge on the folding, stability and function of proteins has tremendously increased over the past years. Moreover, the practical approach of isolating distinct protein domains, while characterizing their properties, has allowed addressing numerous scientific issues that would be too difficult to characterize in a physiological context. Nevertheless, a focused analysis of the role played by the interaction between contiguous domains, disordered tails and quinary structure suggests that there are additional complications to be considered, which demand a careful analysis. In conclusion, whilst the studies on isolated domains represent a valuable advance in knowledge in protein biophysics, we stress the importance to increase the level of complexity and extend the efforts to characterize such domains in more complex contexts.

## Data Availability

Not applicable.
